# Integrated Multi-Omics Analysis Identifies Potential Associations of Unfermented and Fermented Astragalus Straw with Serum Antioxidant Status and Lipid Metabolism in Lambs

**DOI:** 10.3390/ani16193001

**Published:** 2026-09-24

**Authors:** Shulin Liu, Kun Yang, Ruifeng Yang, Huan Shi, Zhixiang Zhao, Yuanyuan Xing

**Affiliations:** 1 Department of Agriculture, Animal Husbandry and Food Science, Hetao College, Bayannur 015000, China; liushulin0723@163.com (S.L.); sh30988@hotmail.com (H.S.); 2 Research Center for Integrated Innovation of Sheep Breeding and Healthy Rearing Technology (HYPT202604), Hetao University, Bayannur 015000, China; 3 College of Animal Science, Inner Mongolia Agricultural University, Hohhot 010018, China; y994454169@163.com; 4 Department of Mechanical and Electrical Engineering, Hetao College, Bayannur 015000, China; yangrf771355@outlook.com (R.Y.); 13015081961@163.com (Z.Z.)

**Keywords:** antioxidant, Astragalus straw, metabolomics, lambs

## Abstract

This study investigated the effects of fermented and unfermented Astragalus straw on serum antioxidant status, lipid metabolism, and hormone profiles in lambs and explored the underlying mechanisms using 16S rRNA sequencing and untargeted metabolomics. Twenty-seven weaned lambs were randomly assigned to three groups: a control group fed a basal total mixed ration (CON), an unfermented Astragalus straw supplementation group (AM_S), and a fermented Astragalus straw supplementation group (FAM_S). The experiment consisted of a 15-day adaptation period followed by a 90-day feeding period. At the end of the trial, growth performance was evaluated and serum samples were collected. Six lambs from each group were randomly selected and euthanized for the collection of rumen fluid and liver tissues for omics analyses. Compared with the CON group, both the AM_S and FAM_S groups showed significantly improved growth performance (*p* < 0.05). Furthermore, glutathione peroxidase (GSH-Px) activity and Adipose triglyceride lipase (ATGL) levels were significantly increased, whereas Low-density lipoprotein cholesterol (LDL) levels, resistin, and insulin (INS) concentrations were significantly decreased in the supplemented groups (*p* < 0.05). Microbiota analysis revealed that g_norank_f_F082 and f_F082 were enriched in the CON group, g_UCG-009 and f_Butyricicoccaceae were enriched in the AM_S group, and f_norank_o_Clostridia_vadinBB60_group and o_Clostridia_vadinBB60_group were enriched in the FAM_S group (*p* < 0.05). Untargeted metabolomics analysis showed that differential metabolites in the AM_S group were enriched in the α-linolenic acid metabolism and vitamin digestion and absorption pathways in both serum and liver samples, with gluconolactone and 9-oxononanoic acid identified as key associated metabolites. Interestingly, metabolites associated with the neuroactive ligand–receptor interaction pathway showed distinct enrichment patterns between serum and liver samples in the FAM_S group, with adenosine and tryptamine identified as key associated metabolites. Correlation analysis further revealed significant associations among serum antioxidant indicators, lipid metabolism-related parameters, hormone levels, differential metabolites, and rumen microbial genera. In conclusion, supplementation with unfermented or fermented Astragalus straw improved antioxidant capacity (GSH-Px) and modulated lipid metabolism-related parameters (ATGL, LDL-C, resistin, and INS) in lambs. These changes were accompanied by alterations in rumen microbiota and serum metabolites, supporting the potential application of Astragalus straw as a functional feed ingredient for ruminants.

## Simple Summary

Dietary supplementation with both unfermented and fermented Astragalus straw improved growth performance, enhanced serum antioxidant capacity, and regulated lipid metabolism in lambs, which may be associated with shifts in rumen microbial communities and metabolites.

## 1. Introduction

After weaning, the rumen of lambs remains underdeveloped, and the functions of various organs are still undergoing maturation [1]. Moreover, the rapid expansion of intensive meat sheep production, along with factors such as high-density housing and prolonged feeding of high-concentrate diets, predisposes lambs to oxidative stress during the fattening period [2]. This condition is often accompanied by impaired lipid metabolism, which increases hepatic metabolic burden, compromises overall health, and restricts growth and development [3]. Previous studies have shown that redox imbalance disrupts serum antioxidant homeostasis, including alterations in key antioxidant enzymes such as superoxide dismutase (SOD) and glutathione peroxidase (GSH-Px) [4]. Meanwhile, abnormal lipid metabolism can promote dysregulated fat deposition and metabolic inflammation [5]. Therefore, oxidative stress and lipid metabolic disorders represent major limiting factors affecting the growth and development of lambs.

Astragalus is a perennial medicinal herb. Its roots have been widely used in traditional medicine due to their various pharmacological activities, including immunomodulatory, antitumor, antioxidant, cardiovascular protective, renal protective, and anti-aging effects [6,7,8]. In China, approximately 92% of Astragalus straw is discarded or openly burned after harvest, generating nearly 300,000 metric tons of agricultural waste annually. This not only results in resource loss but also contributes to environmental pollution [9,10]. Therefore, the efficient utilization of Astragalus straw has become an important research priority. Astragalus straw and its roots contain similar bioactive components, including astragalus polysaccharides, flavonoids, and saponins. However, the high fiber content of Astragalus straw limits its direct application as an animal feed resource [11]. Microbial fermentation provides an effective strategy to improve the nutritional value of crop residues by degrading structural fibers, reducing antinutritional factors, promoting beneficial microbial proliferation, and enhancing feed utilization efficiency [12]. Recent studies have shown that dietary supplementation with Astragalus straw can regulate rumen fermentation characteristics and microbial community composition in meat sheep, thereby improving feed efficiency [13]. Furthermore, fermented Astragalus straw supplementation has been reported to enhance growth performance, serum immune function, and intestinal health in piglets [14]. However, whether fermented or unfermented Astragalus straw can improve growth performance, antioxidant capacity, and lipid metabolism in finishing lambs remains unclear. Therefore, this study hypothesized that dietary supplementation with fermented or unfermented Astragalus straw could improve growth performance and antioxidant capacity and modulate lipid metabolism in lambs. These effects were also associated with changes in rumen microbial community structure and serum metabolites.

The objective of this study was to investigate the effects of fermented and unfermented Astragalus straw supplementation on growth performance, serum antioxidant capacity, and lipid metabolism in finishing lambs. Furthermore, 16S rRNA sequencing and untargeted metabolomics were employed to elucidate the potential mechanisms underlying these effects, providing a theoretical basis for the efficient development and utilization of Astragalus straw as a functional feed resource.

## 2. Materials and Methods

The use of experimental animals was approved by the Animal Protection and Utilization Committee of Inner Mongolia Agricultural University (Approval No. AW02025202-1-3). All animal procedures were conducted in accordance with the National Standard Guidelines for Ethical Review of Animal Welfare (GB/T 35892-2018) [15]. The experiment was conducted from May to August 2025 at a commercial sheep farm in Bayannur City, Inner Mongolia Autonomous Region, China (40°13′–42°28′ N, 105°12′–109°53′ E), with an average ambient temperature of 22 °C. Astragalus straw used in this study was collected from Bayannur City, Inner Mongolia Autonomous Region. The microbial strains used for fermentation, including *Bacillus velezensis*, *Bacillus altitudinis*, and *Lactobacillus buchneri*, were provided by Beijing Zhongnong Dingxin Agriculture and Animal Husbandry Technology Co., Ltd. (Beijing, China). The viable counts of *B. velezensis*, *B. altitudinis*, and *L. buchneri* were ≥2.0 × 10^8^ CFU/g, ≥1.0 × 10^10^ CFU/g, and ≥2.0 × 10^8^ CFU/g, respectively.

For the preparation of fermented Astragalus straw, *B. velezensis*, *B. altitudinis*, and *L. buchneri* were mixed at a ratio of 1:1:2 and inoculated into crushed Astragalus straw. The moisture content was adjusted to 50%, followed by thorough mixing, compaction, and sealing. The mixture was then subjected to anaerobic fermentation at room temperature (20–25 °C) for 45 days before use.

### 2.1. Animals and Experimental Design

Twenty-seven healthy, weaned male Ba Mei lambs (4 months old) with similar body weights (19.1 ± 0.3 kg) were randomly allocated into three groups (*n* = 9 per group): the control group (CON), Astragalus straw supplementation group (AM_S), and fermented Astragalus straw supplementation group (FAM_S), as shown in Table 1. The composition and nutrient levels of the experimental diets are presented in Table 2. The diets were formulated according to the NY/T 816-2004 Standard for Meat Sheep Feeding [16].

All lambs were housed individually in separate pens, with each lamb serving as an experimental unit. Pen temperature was maintained at 18–24 °C under natural lighting and adequate ventilation. The experimental period lasted 105 days, including a 15-day adaptation period and a 90-day feeding period. The nutrient composition of the diets was adjusted according to different fattening stages, and the feeding trial was divided into three phases: early, middle, and late fattening stages. The daily feed allowance was provided in two equal portions at 08:30 and 16:30. All lambs had free access to feed and water throughout the experimental period.

### 2.2. Serum Sample Collection

On day 90 of the feeding trial, blood samples were collected from all lambs in each group via the jugular vein before morning feeding after an overnight fast. Blood samples were collected into 5 mL vacuum tubes without an anticoagulant and allowed to clot at room temperature for 30 min. Subsequently, the samples were centrifuged at 3000× *g* for 15 min to separate the serum. The obtained serum was aliquoted and stored at −20 °C until further analysis.

### 2.3. Rumen Fluid Collection

On day 90 of the feeding trial, six lambs from each group were randomly selected, and rumen fluid samples were collected before morning feeding using a rumen fluid collector connected to a 50 mL syringe. The initial rumen fluid collected was discarded to minimize saliva contamination. Approximately 50 mL of rumen fluid was collected, filtered through four layers of sterile gauze, and stored at −20 °C until 16S rRNA sequencing analysis.

### 2.4. Liver Tissue Collection

On day 90 of the feeding trial, six lambs from each group were randomly selected and slaughtered. Following slaughter, liver samples from the caudate lobe were collected, immediately divided into 2-mL cryovials, and stored in liquid nitrogen until further analysis.

### 2.5. Measurement of Growth Performance

Daily feed intake and feed refusals were recorded for each lamb on an as-fed basis (kg/d). Feed and refusal samples were collected, oven-dried at 105 °C, and analyzed for dry matter (DM) content (%). Individual daily dry matter intake (DMI; kg) was calculated as follows: DMI (kg/d) = feed intake (kg/d) × feed DM fraction − feed refusals (kg/d) × refusal DM fraction. Initial body weight (IBW) and final body weight (FBW) were recorded at the beginning and end of the experiment, respectively. Only the initial and final body weights of lambs in each treatment group were recorded during the formal trial period. Therefore, average daily gain (ADG) was calculated as (FBW − IBW)/90. The feed conversion ratio (F/R) was calculated as DMI (g) divided by ADG (g).

### 2.6. Measurement of Serum Antioxidant, Lipid Metabolism, and Hormone Parameters

Serum antioxidant parameters, including SOD (A001-1-2), catalase (CAT; A007-1-1), GSH-Px (A005-1-2), and malondialdehyde (MDA; A003-1-2), were determined using commercial assay kits. Specifically, SOD activity was measured using the hydroxylamine method, CAT activity using the ammonium molybdate method, GSH-Px activity using a colorimetric method, and MDA content using the thiobarbituric acid (TBA) colorimetric method. All kits were purchased from the Nanjing Jiancheng Bioengineering Institute (Nanjing, China).

Serum lipid metabolism-related parameters were analyzed using commercial assay kits. Fatty acid synthase (FAS; BC0550) activity was determined by UV spectrophotometry, and acetyl-CoA carboxylase (ACC; BC6025) activity was measured using a microassay kit (Solarbio, Beijing, China). Stearoyl-CoA desaturase (SCD; H648-1-1) and adipose triglyceride lipase (ATGL; H305-1-2) levels were determined using enzyme-linked immunosorbent assay (ELISA) kits. Lipoprotein lipase (LPL; A067-1-2) activity was measured using a microassay kit. Total cholesterol (T-CHO; A111-2-1) was determined using a colorimetric assay, high-density lipoprotein cholesterol (HDL-C; A112-1-1) using a direct two-reagent method, and nonesterified fatty acids (NEFA; A042-2-1) and low-density lipoprotein cholesterol (LDL; A113-1-1) using microassay methods. Except for ACC, all kits were purchased from the Nanjing Jiancheng Bioengineering Institute (Nanjing, China).

Serum hormone concentrations, including glucagon (GC; H183-1-2), resistin (H175), insulin (INS; H203-1-1), leptin (LEP; H174-1-2), and adiponectin (ADPN; H179-1-2), were determined using ELISA kits purchased from the Nanjing Jiancheng Bioengineering Institute (Nanjing, China). Insulin-like growth factor-1 (IGF-1; CSB-E13753Sh) was quantified using an ELISA kit purchased from Wuhan Huamei Bioengineering Co., Ltd. (Wuhan, China).

### 2.7. 16S rRNA Sequencing of Rumen Microorganisms

Rumen fluid samples were randomly collected from six lambs in each treatment group and transported to Wuhan Maiwei Metabolic Biotechnology Co., Ltd. (Wuhan, China) for 16S rRNA gene sequencing analysis. Briefly, total DNA was extracted from the rumen fluid samples using the CretMag™ Power Soil DNA Kit (Cretaceous, Suzhou, China). The quality of DNA extraction was assessed by 1% agarose gel electrophoresis, and DNA concentration and purity were determined using a NanoDrop 2000 spectrophotometer (Thermo Scientific, Wilmington, DE, USA). PCR amplification products were pooled and evaluated by electrophoresis on a 2% agarose gel. Qualified PCR products were purified using the AxyPrep DNA Gel Extraction Kit (Axygen Biosciences, Union City, CA, USA). Sequencing libraries were prepared using the VAHTS^®^ Universal Plus DNA Library Prep Kit for MGI V2 (NuoViz, Nanjing, China). After library quality assessment, sequencing was performed on the MGI sequencing platform.

The V3–V4 region of the bacterial 16S rRNA gene was amplified in triplicate using the universal primers 338F (5′-ACTCCTACGGGAGGCAGCAG-3′) and 806R (5′-GGACTACHVGGGTWTCTAAT-3′). PCR was performed in a 20 μL reaction mixture containing 5× FastPfu Buffer, dNTPs, forward and reverse primers, FastPfu polymerase, and template DNA. The amplification program consisted of an initial denaturation at 95 °C for 3 min, followed by 30 cycles of denaturation at 95 °C for 30 s, annealing at 55 °C for 30 s, and extension at 72 °C for 45 s, followed by a final extension at 72 °C for 10 min. PCR products were separated on a 2% agarose gel, purified using the AxyPrep DNA Gel Recovery Kit (Axygen Biosciences, Union City, CA, USA), and quantified using a QuantiFluor™-ST fluorometer (Promega, Madison, WI, USA). Purified amplicons were sequenced on the Illumina PE300 platform.

A total of 9,561,754 raw reads, corresponding to 2,868,526,200 bp, were obtained. After quality filtering and paired-end merging based on overlapping regions, 38,912–111,164 high-quality sequences were retained per sample. The resulting sequences were denoised using DADA2 to generate amplicon sequence variants (ASVs) and their corresponding abundance profiles. Taxonomic classification, microbial community diversity analysis, and differential abundance analysis were subsequently performed based on the ASV representative sequences and abundance data. Taxonomic assignment was conducted using the RDP Classifier against the SILVA database (version 138). DNA extraction blanks and PCR no-template controls were included to monitor potential contamination. ASVs with a total relative abundance of <0.01% were excluded from downstream analyses.

The relative abundance of the top 10 microbial taxa was visualized. Differences in microbial community composition at the phylum and genus levels were analyzed using the Kruskal–Wallis test. Linear discriminant analysis effect size (LEfSe) analysis was performed using LEfSe software (v1.1.0) with an LDA score threshold of 2.5. Spearman correlation analysis was conducted to evaluate the relationships between rumen microbial genera and serum antioxidant capacity, lipid metabolism-related parameters, and hormone levels.

### 2.8. Metabolomic Analysis of Serum and Liver Tissue

Serum and liver tissue samples collected from each treatment group were subjected to untargeted metabolomics analysis. Liver sample preparation: Briefly, 50 mg of liver tissue was accurately weighed and transferred into a 2-mL centrifuge tube containing one grinding bead (6 mm in diameter) and 400 μL of extraction solvent (methanol = 4:1, *v*/*v*). Samples were homogenized using a cryogenic tissue grinder (Wonbio-96c, Shanghai Wanbo Biotechnology Co., Ltd., Shanghai, China) at −10 °C and 50 Hz for 6 min. Subsequently, the samples were extracted by low-temperature ultrasonic treatment at 5 °C and 40 kHz for 30 min using a temperature-controlled ultrasonic cleaner (SBL-10TD, Ningbo Xinzhi Biotechnology Co., Ltd., Ningbo, China). After incubation at −20 °C for 30 min, the samples were centrifuged at 13,000× *g* for 15 min at 4 °C using a refrigerated centrifuge (Centrifuge 5424R, Eppendorf, Hamburg, Germany). The supernatants were transferred into sample vials for subsequent analysis. A pooled quality control (QC) sample was prepared by mixing equal volumes (20 μL) of supernatants from all samples. Serum sample preparation: A 20-μL aliquot of serum from each sample was mixed with 120 μL of 50% methanol, vortexed, and incubated at room temperature for 10 min. The extracts were then stored at −20 °C overnight for protein precipitation and centrifuged at 4 °C and 4000× *g* for 20 min. The resulting supernatants were transferred into 96-well plates for analysis. A pooled QC sample was prepared by mixing equal volumes (10 μL) of supernatants from all samples. All prepared samples were stored at −80 °C until metabolomic analysis. For metabolomic profiling, samples were separated using an Agilent 1290 Infinity II ultra-high-performance liquid chromatography (UHPLC) system(Agilent Technologies, Waldbronn, Germany) and analyzed using a Triple TOF 6600+ mass spectrometer (AB SCIEX, Marlborough, MA, USA). Raw data were processed using XCMS software (v3.12.0) implemented in MetaboAnalyst for peak alignment, retention time correction, and peak area extraction. The extracted data were subjected to metabolite identification, data preprocessing, quality control evaluation, and subsequent statistical analysis. Principal component analysis (PCA) and orthogonal partial least squares discriminant analysis (OPLS-DA) were performed using the R package ropls (version 1.6.2). Model reliability was evaluated using seven-fold cross-validation. Differential metabolites were screened based on the OPLS-DA model with variable importance in projection (VIP) values > 1 and Student’s *t*-test *p* values < 0.05. Metabolites were putatively annotated by matching accurate mass-to-charge ratios and MS/MS fragmentation patterns against reference databases and spectral libraries. HMDB and KEGG were used for metabolite annotation and pathway assignment. No authentic reference standards were used to confirm metabolite identities in the present untargeted metabolomics study. Therefore, the reported metabolite identities should be considered putative annotations rather than unequivocally confirmed identifications. Higher-confidence annotations were assigned when experimentally acquired MS/MS fragmentation spectra showed good agreement with the corresponding reference spectra in the databases or spectral libraries. Quantitative analysis of identified metabolites was performed using the metaX software (v1.4.2). Spearman correlation analysis was conducted to investigate the associations between differential metabolites involved in commonly enriched pathways in serum and liver samples and the corresponding rumen microbial genera across treatment groups. Multiple comparisons were corrected using the Benjamini–Hochberg false discovery rate (BH-FDR) method. Spearman’s correlation coefficients and corresponding *p*-values were calculated between bacterial genera and metabolites across all samples from the CON, AM_S, and FAM_S groups, resulting in 132 correlation tests. Significant correlations were defined as |r| > 0.5 and an FDR-adjusted *p*-value < 0.05.

### 2.9. Statistical Analysis

Statistical analyses were performed using SPSS software (version 27.0). One-way analysis of variance (ANOVA) was conducted to evaluate differences among treatment groups. Pairwise comparisons were performed, and the resulting *p*-values were adjusted using the Holm–Bonferroni method to control the family-wise error rate. Data are presented as means ± standard error of the mean (SEM), and differences were considered statistically significant when the adjusted *p* < 0.05.

## 3. Results

### 3.1. Effects of Fermented and Unfermented Astragalus Straw on Growth Performance

As shown in Table 3, compared with the CON group, both the AM_S and FAM_S groups exhibited significantly increased FBW (*p* = 0.013), TWG (*p* = 0.020), and ADG (*p* = 0.020), along with a significantly reduced F/G in lambs (*p* = 0.014). Notably, the FAM_S group showed a significantly higher DMI compared with both the CON and AM_S groups (*p* = 0.003).

### 3.2. Effects of Fermented and Unfermented Astragalus Straw on Serum Antioxidant Parameters

As shown in Table 4, compared with the CON group, the AM_S and FAM_S groups showed no significant differences in serum SOD (*p* = 0.843) and CAT (*p* = 0.524) activities or MDA levels in lambs (*p* = 0.328). However, GSH-Px activity was significantly increased in both the AM_S and FAM_S groups (*p* = 0.003), with no significant difference observed between these two groups (*p* = 0.951).

### 3.3. Effects of Fermented and Unfermented Astragalus Straw on Serum Lipid Metabolism Parameters

As shown in Table 5, compared with the CON group, no significant differences were observed in serum FAS activity (*p* = 0.928) or HDL-C levels (*p* = 0.065) in the AM_S group. In contrast, the FAM_S group exhibited significantly decreased FAS activity (*p* = 0.048) and increased HDL-C levels (*p* = 0.047). Compared with the CON group, both the AM_S and FAM_S groups showed significantly increased ATGL activity in serum (*p* = 0.001).

### 3.4. Effects of Fermented and Unfermented Astragalus Straw on Serum Hormone Levels

As shown in Table 6, compared with the CON group, the FAM_S group exhibited significantly increased serum GC levels in lambs (*p* = 0.001). In addition, both the AM_S and FAM_S groups showed significantly decreased serum resistin (*p* = 0.005) and INS concentrations compared with the CON group (*p* = 0.002).

### 3.5. 16S rRNA Sequencing Analysis of Rumen Microbiota

To investigate the effects of fermented and unfermented Astragalus straw supplementation on rumen microbiota composition in lambs, 16S rRNA sequencing was performed on rumen fluid samples collected from the CON, AM_S, and FAM_S groups. The results showed no significant differences in alpha diversity indices, including the Chao1, Shannon, Simpson, and Sobs indices, among the three groups (Table S1). As shown in the PCoA plot, the CON, AM_S, and FAM_S groups were clearly separated. ANOSIM further confirmed significant differences in rumen bacterial community structure among the three groups (R = 0.315, *p* = 0.001; Figure 1A). Shared amplicon sequence variants (ASVs) among the CON, AM_S, and FAM_S groups were further visualized using a Venn diagram (Figure 1B).

Analysis of the top 15 dominant microbial taxa revealed that *Bacillota*, *Bacteroidota*, *Thermodesulfobacteriota*, and other phyla exhibited high relative abundances at the phylum level across all groups. At the genus level, *norank_f_F082*, *Rikenellaceae_RC9_gut_group*, *norank_f_Muribaculaceae*, and other genera were among the predominant taxa in the rumen microbiota (Figure 1C,D).

Compared with the CON group, the relative abundances of *norank_f_F082* (*p* = 0.015), *NK4A214_group* (*p* = 0.022), *[Eubacterium]_ventriosum_group* (*p* = 0.004), and other genera were significantly decreased in both the AM_S and FAM_S groups; moreover, this effect was more pronounced in the FAM_S group (Figure 2A).

LEfSe analysis was subsequently performed to identify discriminatory microbial taxa among the treatment groups (LDA score > 2.5, *p* < 0.05; Figure 2B,C). A total of 22 bacterial taxa were significantly enriched among the three groups. In the CON group, *g_norank_f_F082* (*p* = 0.015), *f_F082* (*p* = 0.015), *g_NK4A214_group* (*p* = 0.022), and other taxa were significantly enriched. In the AM_S group, *g_UCG-009* (*p* = 0.017), *f_Butyricicoccaceae* (*p* = 0.018), *g_Prevotellaceae_UCG-004* (*p* = 0.035), and other taxa were significantly enriched (*p* < 0.05). In the FAM_S group, *f_norank_o_Clostridia_vadinBB60_group* (*p* = 0.015), *o_Clostridia_vadinBB60_group* (*p* = 0.023), *g_norank_o_Clostridia_vadinBB60_group* (*p* = 0.031), and other taxa were significantly enriched.

To investigate the potential associations between rumen microbiota and serum antioxidant and lipid metabolism parameters, Spearman correlation analysis was performed between differential microbial genera and serum biochemical indicators. After multiple-testing correction using the BH-FDR method, none of the correlations met the predefined significance criteria (|r| > 0.5 and FDR-adjusted *p* < 0.05). Therefore, no significant associations between differential bacterial genera and serum biochemical parameters were identified after correction for multiple testing.

Exploratory analysis based on unadjusted *p*-values identified several nominal associations (raw *p* < 0.05). For example, *g_Tractidigestivibacter* and *g_Prevotellaceae_UCG-004* were positively correlated with GSH-Px activity. In addition, *g_Roseburia* and *g_NK4A214_group* were negatively correlated with serum FAS and GC levels, whereas *g_Roseburia* was positively correlated with ATGL, LDL-C, resistin, and INS levels. However, none of these associations remained significant after FDR correction and should therefore be interpreted as exploratory findings.

### 3.6. Metabolomic Analysis of Serum and Liver

In this study, untargeted metabolomics analysis was performed on serum and liver tissue samples collected from weaned lambs in the different treatment groups. PCA illustrated the distribution of serum metabolic profiles across the CON, AM_S, and FAM_S groups, with partial overlap among samples. ANOSIM detected significant differences among the groups (R = 0.260, *p* = 0.001). In contrast, PCA of liver metabolic profiles showed substantial overlap among samples, and ANOSIM detected no significant differences among the three groups (R = 0.031, *p* = 0.323).

Based on metabolite classification, the identified metabolites in both serum and liver samples were mainly categorized into lipids and lipid-like molecules, organic acids and derivatives, organoheterocyclic compounds, organic oxygen compounds, and benzenoids (Figure 3B). The numbers of upregulated and downregulated differential metabolites among the treatment groups are shown in Figure 3C, indicating that fermented and unfermented Astragalus straw supplementation exerted distinct regulatory effects on metabolite profiles in lamb serum and liver. Furthermore, orthogonal partial least squares discriminant analysis (OPLS-DA) combined with variable importance in projection (VIP) analysis was applied to identify the top 30 differential metabolites in serum and liver tissues among the treatment groups (Figure S1).

Subsequently, KEGG pathway enrichment analysis was performed to investigate the metabolic pathways associated with differential metabolites in serum and liver samples from each treatment group (Figure 4). Compared with the CON group, the differential metabolites commonly enriched in both serum and liver of lambs in the AM_S group were primarily involved alpha-linolenic acid metabolism, vitamin digestion and absorption, and the pentose phosphate pathway (Figure 4A). These alterations were mainly associated with gluconolactone, 9-oxo-nonanoic acid, and flavin mononucleotide in serum, as well as 2,5-diamino-6-(5-phospho-D-ribosylamino)-pyrimidin-4(3H)-one, pyridoxal phosphate, and alpha-linolenic acid in liver tissue (Figure 4B). Compared with the CON group, the differential metabolites in the FAM_S group were commonly enriched in the neuroactive ligand–receptor interaction pathway in both serum and liver (Figure 4C). This alteration was primarily associated with adenosine, tryptamine, and sphingosine 1-phosphate in serum and prostaglandin E2, L-glutamic acid, and palmitoylethanolamide in liver tissue (Figure 4D).

No commonly enriched pathways were identified between serum and liver differential metabolites when comparing the FAM_S and AM_S groups (Figure 4E). However, compared with unfermented Astragalus straw supplementation, fermented Astragalus straw supplementation mainly enriched benzoxazinoid biosynthesis in serum, with indole identified as the key differential metabolite involved in this pathway. In liver tissue, fermented Astragalus straw primarily enriched fatty acid degradation, with palmitoylcarnitine identified as the major differential metabolite associated with this pathway (Figure 4E,F).

### 3.7. Correlation Analysis Between Rumen Microbiota and Serum and Liver Metabolites

Subsequently, Spearman’s correlation analysis was performed between these microbial genera and differential metabolites involved in signaling pathways commonly enriched in both serum and liver samples (Figure 5).

The results demonstrated that differential metabolites exhibited significant correlations with multiple rumen microbial genera (|r| > 0.5 and FDR-adjusted *p* < 0.05). For instance, compared with the CON group, flavin mononucleotide in the serum of lambs in the AM_S group showed a significant negative correlation with *g_[Eubacterium]_ventriosum_group* (r = −0.850, *p* = 0.021; Figure 5A). In liver tissue, 2,5-diamino-6-(5-phospho-D-ribosylamino)pyrimidin-4(3H)-one was negatively correlated with *g_Tannerella* (r = −0.702, *p* = 0.035), whereas riboflavin showed a significant positive correlation with *g_Prevotellaceae_UCG-004* (r = 0.810, *p* = 0.012; Figure 5A). Compared with the CON group, serum tryptamine levels in the FAM_S group exhibited a significant negative correlation with *g_UCG-009* (r = −0.960, *p* = 0.034), while adenosine showed a significant positive correlation with *g_Roseburia* (r = 0.577, *p* = 0.035; Figure 5B). Additionally, palmitoylethanolamide in the liver tissue of lambs from the FAM_S group was positively correlated with *g_Tannerella* (r = 0.654, *p* = 0.041; Figure 5B).

## 4. Discussion

The bioactive compounds present in Astragalus, including Astragalus polysaccharides and flavonoids, have been reported to improve animal growth performance, enhance antioxidant capacity, modulate gut microbiota composition, and promote intestinal health [17,18,19]. Astragalus straw also contains various pharmacologically active components [20], suggesting its potential as a functional feed resource to improve animal productivity and health. Previous studies have demonstrated that fermentation can degrade structural carbohydrates in crop straw, reduce pH, increase crude protein and lactic acid contents, and consequently improve palatability and nutritional value [21,22]. In the present study, dietary supplementation with both unfermented and fermented Astragalus straw improved growth performance and feed efficiency in lambs. Notably, DMI was significantly higher in the FAM_S group than in the CON and AM_S groups. Therefore, the improved growth performance may have resulted partly from increased energy and protein intake rather than solely from the bioactive components of fermented Astragalus straw. Potential confounding factors, including dietary composition, the fiber-to-starch balance, and feed palatability, should also be considered. Therefore, the present experimental design cannot fully distinguish the effects of fermented Astragalus straw itself from those associated with increased feed intake. Interestingly, serum GSH-Px activity was significantly increased in both the AM_S and FAM_S groups. Consistently, Li et al. [23] reported that dietary Astragalus polysaccharides enhanced growth performance and increased serum GSH-Px activity in dairy cows. Similarly, Luo et al. [24] demonstrated that Astragalus supplementation increased the activities of antioxidant enzymes, including SOD, CAT, and GSH-Px, in muscle tissue of cashmere goats, thereby improving antioxidant capacity. In summary, these findings indicate that both unfermented and fermented Astragalus straw enhances the growth performance of lambs regardless of fermentation status. It also improves antioxidant capacity by increasing serum GSH-Px levels, potentially through the antioxidant activity of bioactive compounds such as Astragalus polysaccharides.

FAS is a key enzyme involved in de novo fatty acid synthesis and promotes endogenous fatty acid production [25], whereas ATGL plays a crucial role in lipolysis and maintaining lipid metabolism homeostasis [26]. Oxidative stress and inflammatory injury can disrupt lipid metabolism, characterized by decreased HDL-C levels and elevated LDL levels, thereby increasing the risk of lipid accumulation and metabolic disorders [27]. Yao et al. [28] reported that Astragalus isoflavones improved dyslipidemia and reduced hepatic lipid accumulation in mice. Liu et al. [29] further demonstrated that fermented Citri Sarcodactylis Fructus exerted stronger effects than unfermented Citri Sarcodactylis Fructus in improving insulin resistance, lipid metabolism disorders, oxidative stress, and inflammation, which may be attributed to the enhanced availability of bioactive compounds, such as flavonoids, after fermentation. In the present study, supplementation with both unfermented and fermented Astragalus straw increased serum ATGL activity and reduced LDL levels in lambs. Moreover, fermented Astragalus straw supplementation significantly decreased FAS activity and increased HDL-C levels. However, this study did not characterize changes in the bioactive components of fermented Astragalus straw. Therefore, the specific bioactive compounds that may have contributed to the observed effects remain unclear.

Hormonal regulation also plays an important role in lipid metabolism. INS promotes fatty acid synthesis and inhibits lipolysis, whereas GC stimulates gluconeogenesis and facilitates the mobilization of stored energy. Conversely, resistin can induce insulin resistance by impairing insulin signaling, thereby contributing to lipid metabolism disorders and oxidative stress [30,31,32]. In this study, supplementation with both unfermented and fermented Astragalus straw decreased resistin and INS concentrations in lambs, with fermented Astragalus straw showing a stronger effect on enhancing GC levels. Previous studies have demonstrated that fermentation of crop residues can effectively degrade structural carbohydrates, improve nutrient availability, and enhance palatability [33,34]. Moreover, fermented crop straw can modulate rumen microbial community composition in ruminants [35] and enhance the bioavailability and biological activity of functional compounds [36]. However, it remains unclear whether fermentation altered the bioactive components of Astragalus straw in this study. Further investigation is therefore needed to characterize these changes.

Bioactive compounds derived from traditional Chinese herbal medicines have been reported to modulate gut microbiota composition, thereby improving growth performance and antioxidant capacity in lambs [37]. Li et al. [38] demonstrated that Astragalus polysaccharides reduced the relative abundances of *Rikenellaceae_RC9_gut_group* and *unclassified_Prevotellaceae* in the rumen of lambs, which was associated with improved meat quality and immune function. Similarly, Shao et al. [39] reported that dietary supplementation with Astragalus powder increased the relative abundances of *Butyrivibrio fibrisolvens*, *Ruminococcus flavefaciens*, *Succiniclasticum*, and *Prevotella* in the rumen of lambs, contributing to the regulation of lipid metabolism. In the present study, rumen fluid samples from lambs in different treatment groups were analyzed using 16S rRNA sequencing to evaluate the effects of Astragalus straw supplementation before and after fermentation on rumen microbial composition. The results demonstrated that both unfermented and fermented Astragalus straw significantly altered the rumen microbial community structure. Specifically, the relative abundances of *norank_f_F082* and *NK4A214_group* were significantly decreased following Astragalus straw supplementation, with more pronounced reductions observed in the FAM_S group. Zhang et al. [40] reported that the relative abundance of *norank_f_F082* was significantly increased in the rumen of dairy goats suffering from ruminal acidosis. Similarly, Liu et al. [41] demonstrated that increased abundance of *NK4A214_group* in the human gut was associated with elevated inflammatory cytokine levels and chronic inflammatory responses; however, the functional significance of this microbial group in ruminants remains largely unexplored. Subsequently, LEfSe analysis (LDA score > 2.5, *p* < 0.05) was performed to identify rumen microbial taxa enriched in each treatment group. Spearman’s correlation analysis was further conducted to evaluate the associations between differential rumen microbial genera and serum parameters, lipid metabolism enzymes, and hormone indicators. However, none of these associations remained significant after FDR correction and should therefore be interpreted as exploratory findings. Exploratory analysis based on unadjusted *p*-values identified several nominal associations. The results revealed correlations between several rumen microbial genera and GSH-Px activity. For example, *g_Tractidigestivibacter* and *g_Prevotellaceae_UCG-004* were positively correlated with GSH-Px activity. Consistently, Luo et al. [42] reported that the abundance of *g_Prevotellaceae_UCG-001* was positively associated with intestinal antioxidant capacity in Yangzhou geese. Moreover, Li et al. [43] demonstrated that *g_Prevotellaceae_UCG-001* was associated with reduced fattening duration and a lower risk of ruminal acidosis in sheep. Furthermore, *g_Roseburia*, and *g_NK4A214_group* exhibited correlations with several lipid metabolism-related enzymes and hormone indicators. Specifically, *g_Roseburia* and *g_NK4A214_group* were negatively correlated with serum FAS and GC levels, whereas *g_Roseburia* was positively correlated with ATGL, LDL-C, resistin, and INS levels. Previous studies have reported that *g_Roseburia* abundance is negatively associated with serum triglyceride levels and may alleviate high-fat diet-induced disorders of glucose and lipid metabolism and hepatic steatosis in mice [44]. However, current evidence regarding *g_Tannerella* is primarily limited to its association with periodontal disease in humans [45], and its functional role in ruminants remains unclear. Therefore, further studies are required to elucidate the potential mechanisms underlying the relationship between these rumen microbial taxa and lipid metabolism regulation in lambs.

Traditional Chinese medicinal herbs and their bioactive components can influence liver health by modulating gut microbiota-derived metabolites [46,47]. In ruminants, non-carbohydrate substrates generated through rumen microbial digestion and metabolism are primarily converted into glucose through hepatic gluconeogenesis to meet energy requirements [48]. Moreover, the liver plays a central role in maintaining carbohydrate and lipid metabolic homeostasis in ruminants [49]. Non-targeted metabolomics analyses were performed on serum and liver samples from lambs in each treatment group, followed by Spearman’s correlation analysis between differential metabolites and rumen microbial genera. The results demonstrated that, compared with the CON group, the differential metabolites co-enriched in both serum and liver samples of lambs in the AM_S group were primarily associated with riboflavin metabolism and alpha-linolenic acid metabolism. This pathway was primarily associated with increased flavin mononucleotide levels in serum and D-glucosaminic acid levels in the liver. Furthermore, differential metabolites involved in this pathway exhibited significant correlations with multiple rumen microbial genera. Previous studies have demonstrated that increased levels of metabolites such as flavin mononucleotide are associated with riboflavin metabolism, which may contribute to the maintenance of lipid metabolic homeostasis in periparturient dairy cows [50] and improved antioxidant capacity in juvenile turbot [51]. In addition, alpha-linolenic acid metabolism has been reported to reduce serum triglyceride levels and enhance antioxidant capacity [52]. In the present study, compared with the CON group, the predominant pathway jointly enriched by differential metabolites in serum and liver samples of lambs in the FAM_S group was the “Neuroactive ligand–receptor interaction” pathway. This pathway was associated with levels of metabolites such as tryptamine and sphingosine 1-phosphate in serum, along with levels of metabolites such as prostaglandin E2 and L-glutamic acid in the liver. Furthermore, differential metabolites involved in this pathway exhibited significant correlations with multiple rumen microbial genera. Previous studies have shown that catalase supplementation enhances antioxidant capacity by reducing the levels of metabolites such as tryptamine, putrescine, cadaverine, and spermidine in the intestine of weaned rabbits [53]. However, whether these metabolites exert similar regulatory effects on antioxidant status and lipid metabolism in ruminants remains largely unknown, further studies are required to validate the functional roles of these key metabolites in lambs. Furthermore, although some studies have shown that gastrointestinal microbiota can influence the host by producing secondary metabolites [46,47], the origin of the metabolites identified in this study remains unclear. Specifically, it is unknown whether these metabolites originated from the diet, host tissues, or the lambs’ rumen microbiota. Additionally, no a priori sample size or power calculation was performed in this study. Considering experimental costs and animal welfare, only six lambs per group were included in the multi-omics analyses. Therefore, the relatively small sample size may limit the generalizability of the findings, and further studies with larger sample sizes are needed to validate these results.

Interestingly, the present study also revealed that although unfermented and fermented Astragalus straw shared common enriched pathways in serum and liver samples, the specific differential metabolites involved in these pathways varied between treatments. The underlying mechanisms responsible for these differences remain unclear and warrant further investigation. Furthermore, a limitation of this study is that the composition of the fermented Astragalus straw was not characterized. Therefore, changes in its nutrient composition and bioactive compounds, as well as their potential effects on lambs, remain unclear. Further studies are needed to characterize changes in nutrients, bioactive compounds, and microbial communities during the fermentation of Astragalus straw.

## 5. Conclusions

In summary, both unfermented and fermented Astragalus straw supplementation improved antioxidant status (GSH-Px) and modulated lipid metabolism (ATGL, LDL, resistin, and INS) in lambs. Integrated analyses of 16S rRNA sequencing and untargeted metabolomics suggested that these beneficial effects may be associated with alterations in rumen microbial composition and changes in multiple differential serum metabolites. Furthermore, comparison of the commonly enriched pathways in serum and liver revealed that unfermented and fermented Astragalus straw may exert their effects through distinct metabolic regulatory mechanisms.

## Figures and Tables

**Figure 1:**
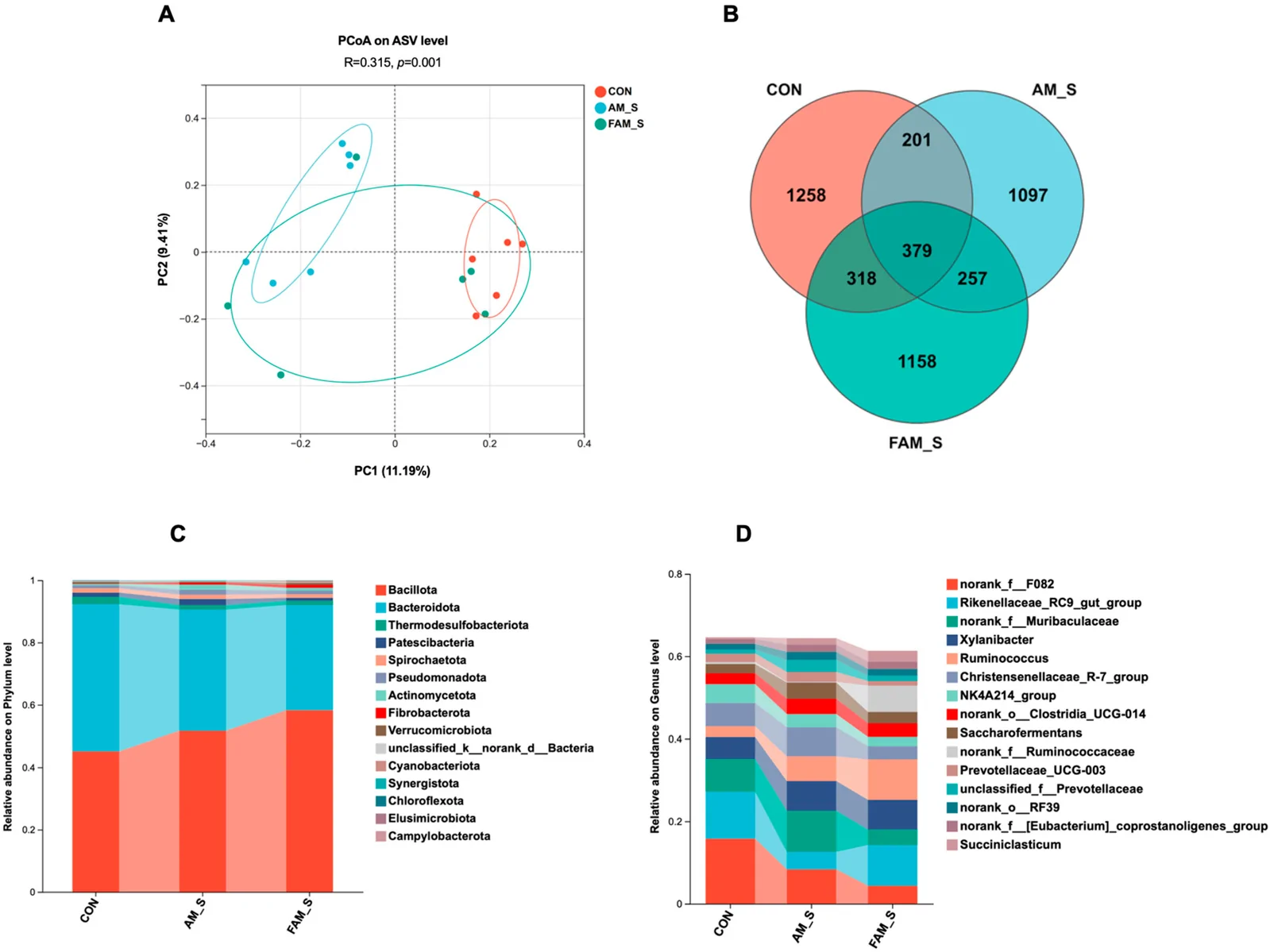
Changes in rumen microbial community structure among lambs from different treatment groups (*n* = 6). (**A**) Principal coordinate analysis (PCoA) based on the weighted UniFrac distance metric showing the beta diversity of bacterial amplicon sequence variants (ASVs) in rumen fluid samples. (**B**) Venn diagram illustrating the numbers of unique and shared ASVs among the three groups. (**C**,**D**) Relative abundance of rumen microbiota composition at the phylum (**C**) and genus (**D**) levels. CON, basal diet; AM_S, basal diet supplemented with Astragalus straw; FAM_S, basal diet supplemented with fermented Astragalus straw.

**Figure 2:**
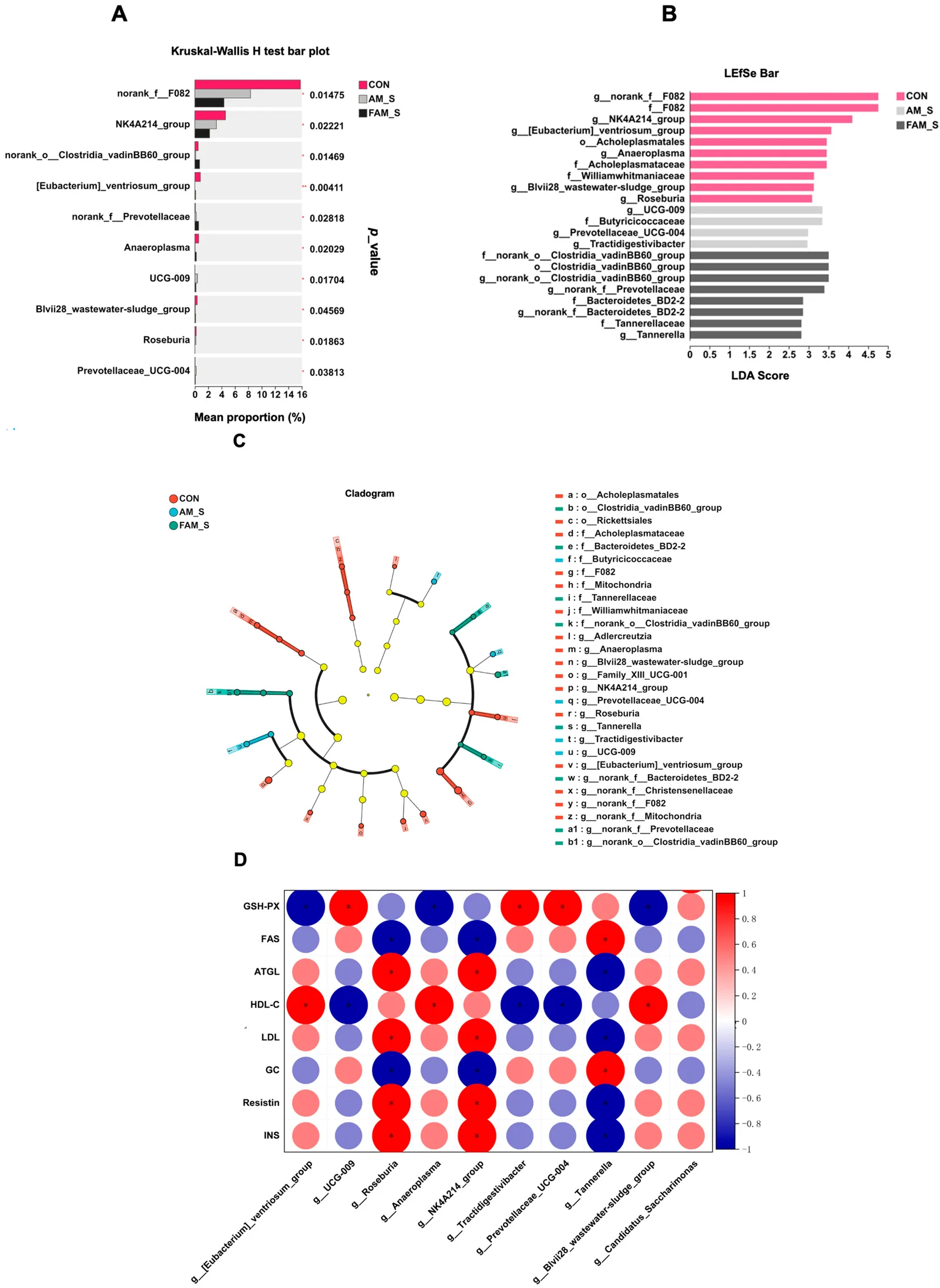
LEfSe and Spearman correlation analyses of rumen microbial communities in lambs from different treatment groups (*n* = 6). (**A**) Kruskal–Wallis H test bar plot showing the mean relative abundance of significantly different bacterial genera among the three groups. The values on the right indicate the corresponding *p*-values. (**B**) LEfSe bar plot showing differentially abundant bacterial taxa; the horizontal axis represents the linear discriminant analysis (LDA) score. (**C**) LEfSe cladogram illustrating the phylogenetic distribution of discriminative taxa. Yellow nodes indicate taxa that did not differ significantly among groups. Red, light blue, and green nodes indicate taxa enriched in the CON, AM_S, and FAM_S groups, respectively. (**D**) Bubble heatmap showing Spearman correlation analysis between differential bacterial genera and serum physiological parameters. Red and blue circles represent positive and negative correlations, respectively. The color intensity and circle size indicate the correlation coefficient. Asterisks denote statistically significant correlations (*p* < 0.05). GSH-Px, glutathione peroxidase; FAS, fatty acid synthase; ATGL, adipose triglyceride lipase; HDL-C, high-density lipoprotein cholesterol; LDL, low-density lipoprotein cholesterol; GC, glucagon; INS, insulin. Six biological replicates were included per treatment group. CON, basal diet; AM_S, basal diet supplemented with Astragalus straw; FAM_S, basal diet supplemented with fermented Astragalus straw. Correlations shown in (**D**) were based on unadjusted *p*-values and did not remain significant after FDR correction.

**Figure 3:**
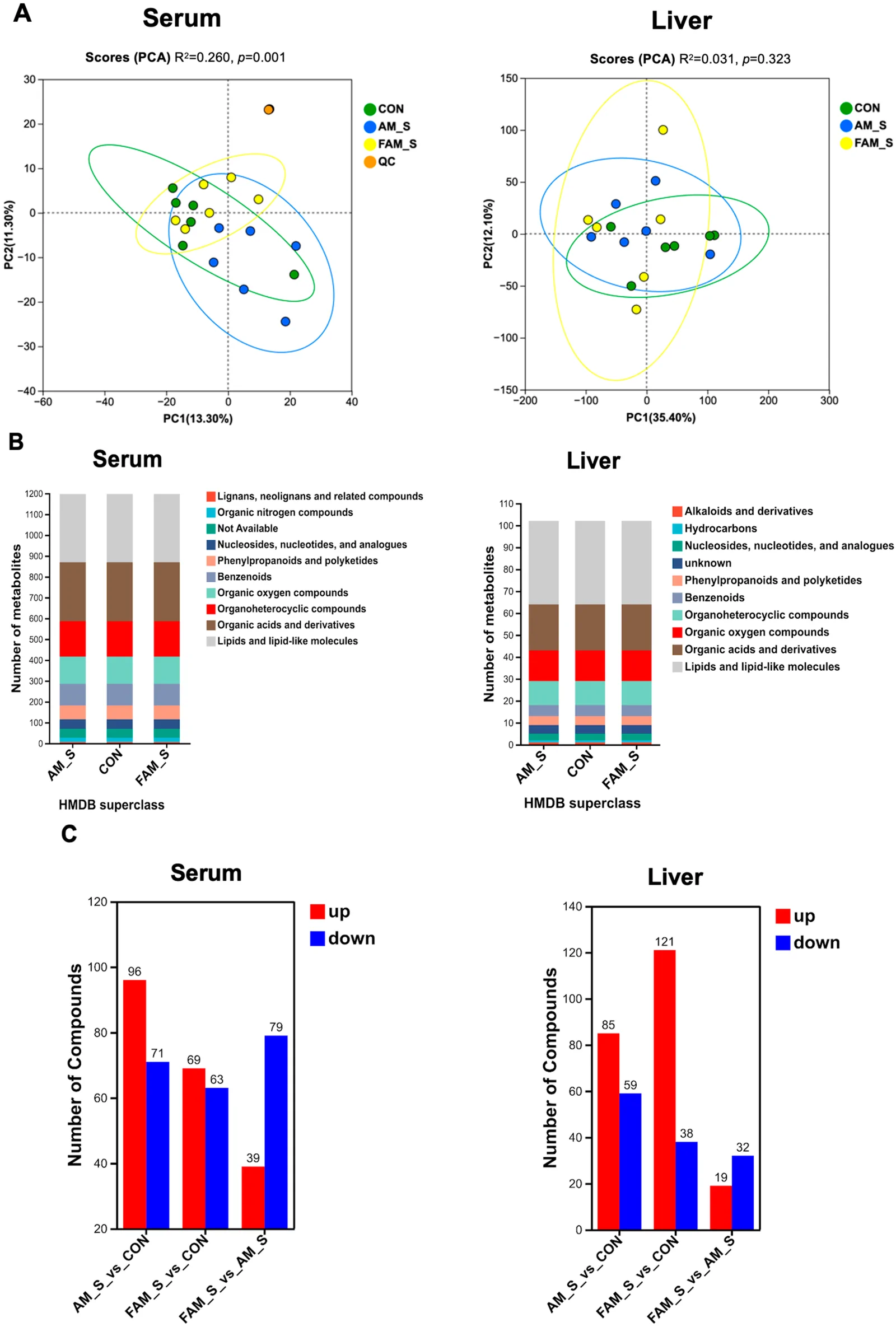
Untargeted metabolomics analysis of serum and liver tissues from lambs in different treatment groups (*n* = 6). (**A**) Principal component analysis (PCA) score plots of metabolite profiles in serum and liver samples. Quality control (QC) samples were included to assess the stability and reproducibility of the analytical platform. The R^2^ values and *p*-values were calculated using ANOSIM analysis. (**B**) Classification of identified metabolites according to the Human Metabolome Database (HMDB) superclass. (**C**) Number of significantly upregulated (red) and downregulated (blue) differential metabolites identified in pairwise comparisons among treatment groups. CON, basal diet; AM_S, basal diet supplemented with Astragalus straw; FAM_S, basal diet supplemented with fermented Astragalus straw.

**Figure 4:**
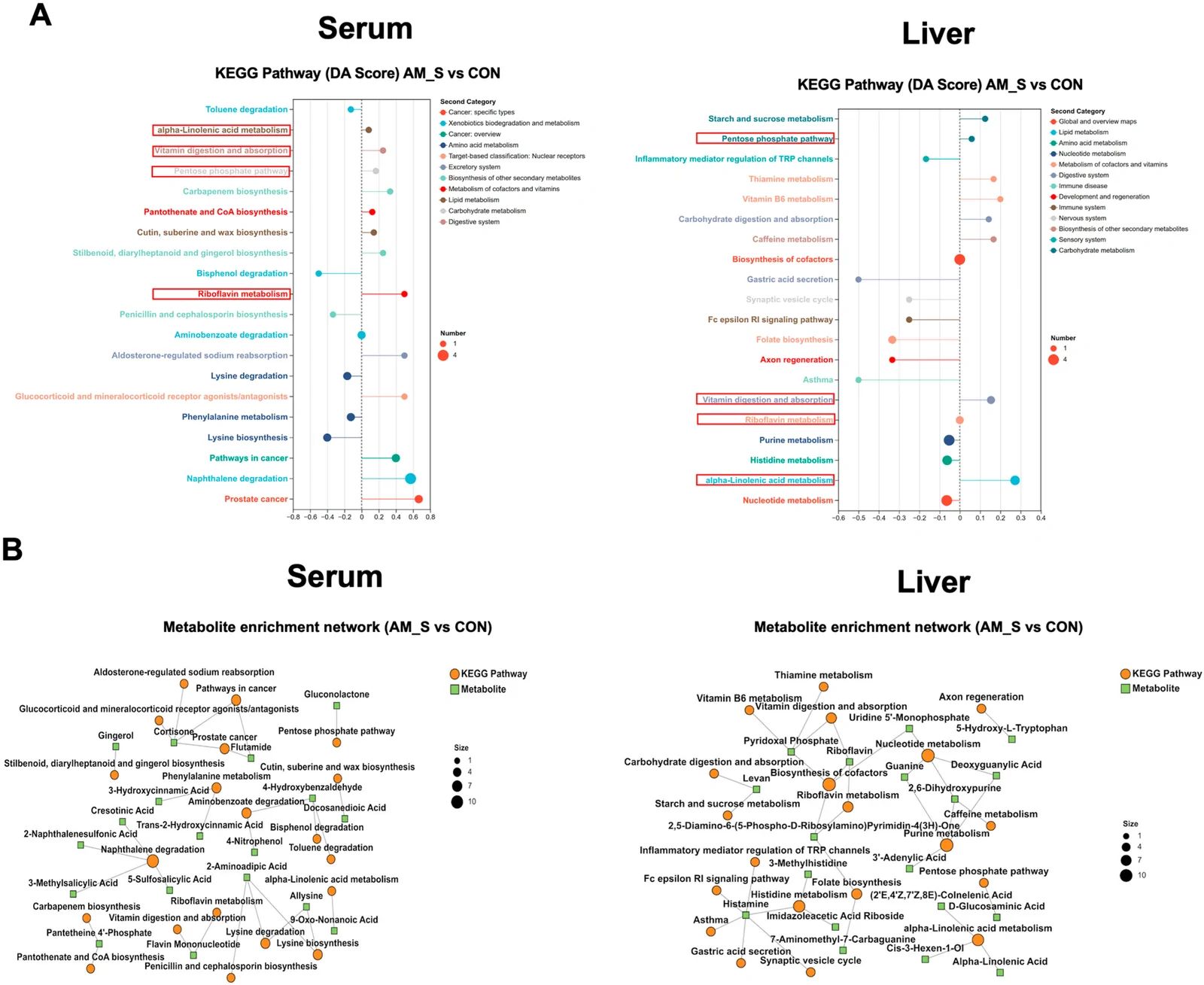

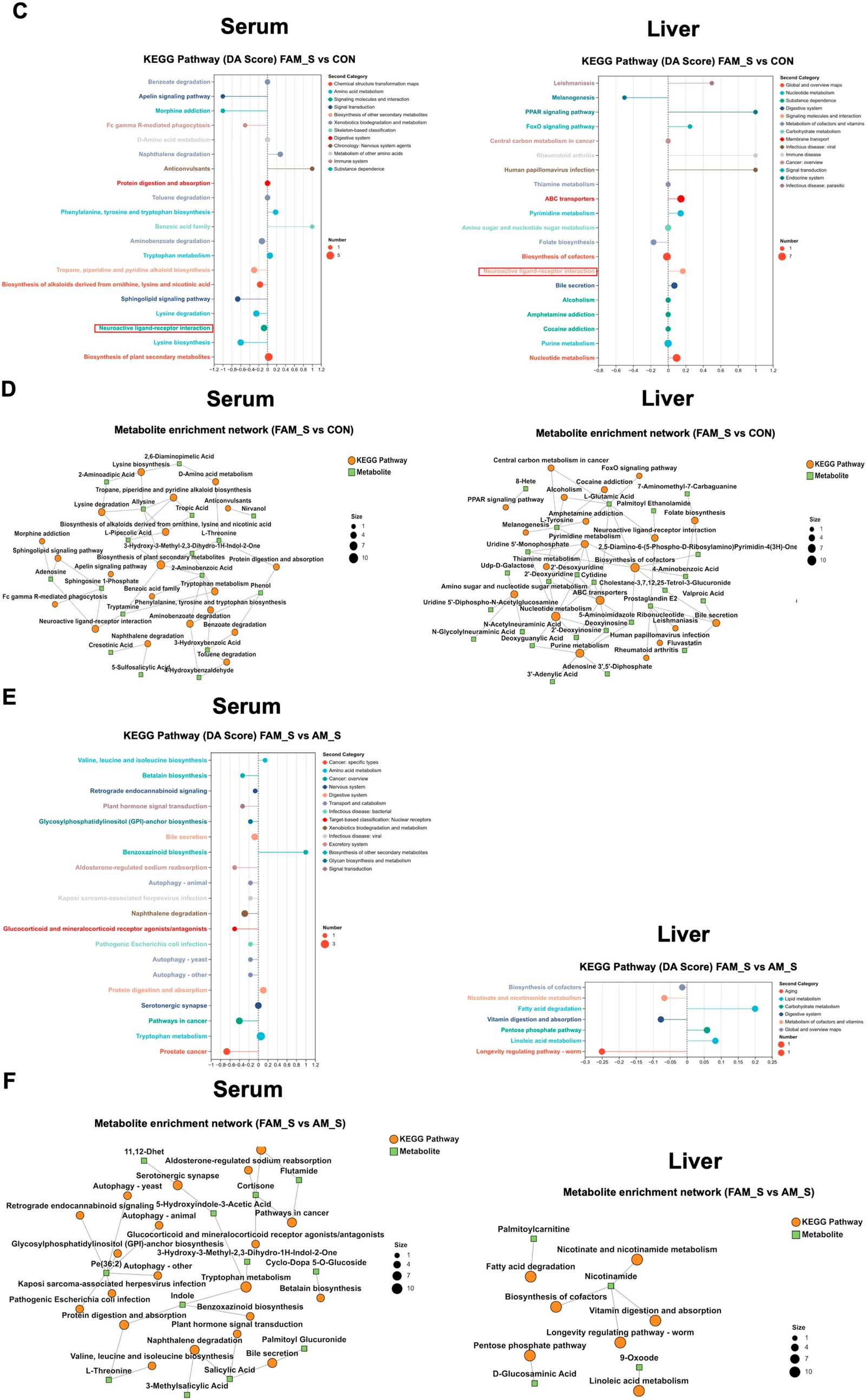
KEGG enrichment analysis of differential metabolites (*n* = 6). (**A**,**C**,**E**) KEGG pathway enrichment bubble plots (DA score) of differential metabolites identified in serum and liver samples. The horizontal axis represents the DA score; dot size indicates the number of differential metabolites enriched in each pathway, and dot color represents different KEGG pathway secondary categories. (**B**,**D**,**F**) Metabolite–pathway enrichment networks of serum and liver differential metabolites. Orange circles represent KEGG pathways, and green squares represent differential metabolites. Node size indicates the number of differential metabolites associated with each pathway. CON, basal diet; AM_S, basal diet supplemented with Astragalus straw; FAM_S, basal diet supplemented with fermented Astragalus straw.

**Figure 5:**
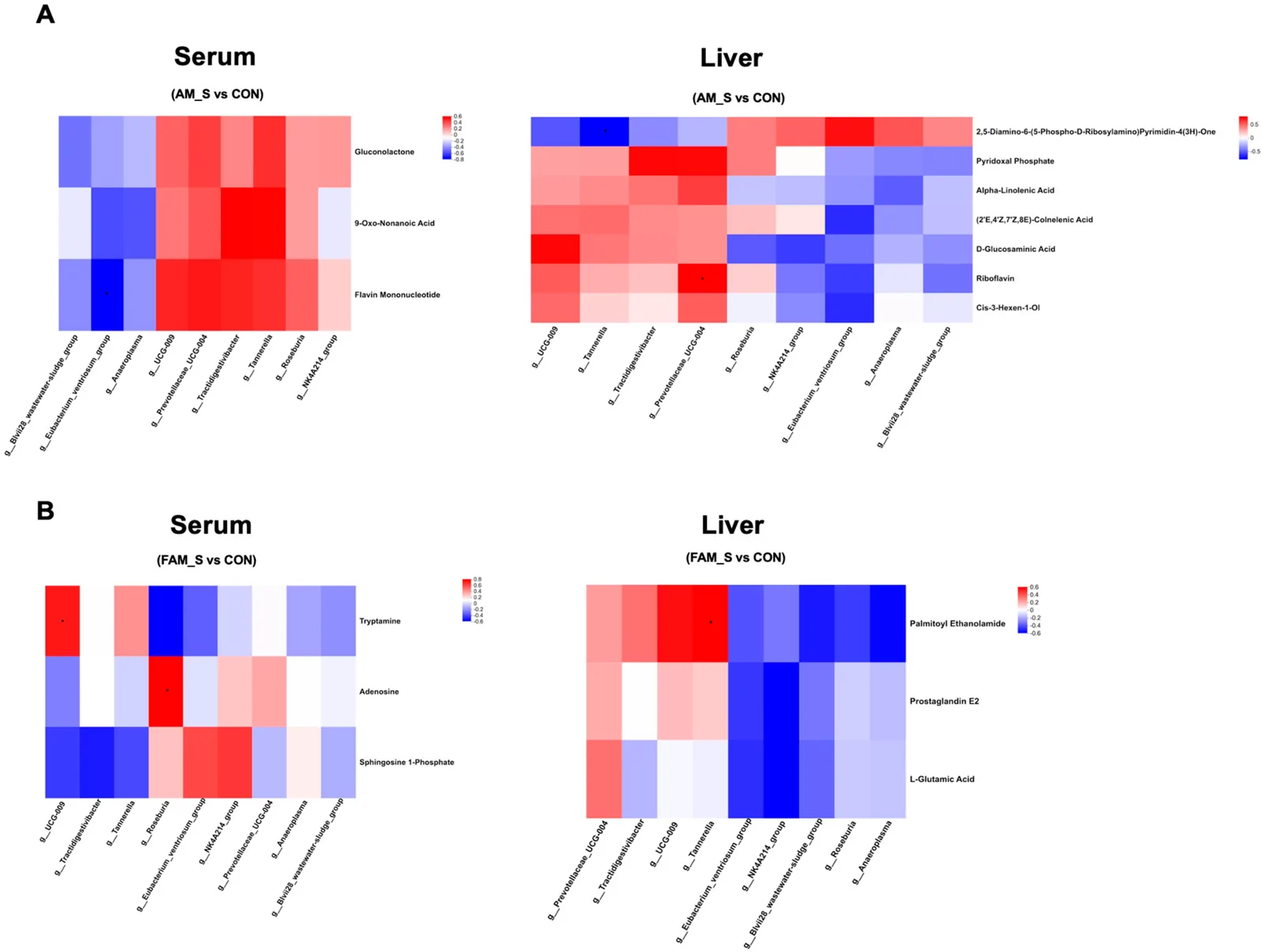
Correlation heatmap between differential rumen bacterial genera and differential metabolites in serum and liver samples. (**A**) Correlations between gut microbial genera and serum/liver metabolites for AM_S relative to CON. (**B**) Correlations between gut microbial genera and serum/liver metabolites for FAM_S relative to CON. The color gradient represents the Spearman correlation coefficient (red indicates positive correlation, whereas blue indicates negative correlation). Asterisks denote statistically significant correlations. CON, basal diet; AM_S, basal diet supplemented with Astragalus straw; FAM_S, basal diet supplemented with fermented Astragalus straw.

**Table 1:** Experiment design.

Group	Number of Lambs	Daily Ration
Control (CON)	9	Basic Diet (TMR)
Astragalus straw (AM_S)	9	Basal diet (TMR) supplemented with Astragalus straw (Astragalus straw partially replacing corn stover and sunflower hulls).
Fermented astragalus Straw (FAM_S)	9	Basal diet (TMR) supplemented with fermented Astragalus straw (Astragalus straw replacing part of the diet on a dry matter basis).

**Table 2:** The composition and nutrient levels of CON, AM_S and FAM_S groups (dry-matter basis, DM basis).

Ingredients, %	Day 1 to 30			Day 31 to 60			Day 61 to 90		
	CON	AM_S	FAM_S	CON	AM_S	FAM_S	CON	AM_S	FAM_S
Corn stalk	16.00	9.00	9.00	16.00	9.00	9.00	16.00	10.00	10.00
Sunflower hulls	16.00	9.00	9.00	16.00	9.00	9.00	16.00	12.00	12.00
Astragalus straw	0.00	14.00	0.00	0.00	14.00	0.00	0.00	10.00	0.00
Fermented astragalus straw	0.00	0.00	14.00	0.00	0.00	14.00	0.00	0.00	10.00
Corn	36.00	39.00	39.00	36.00	39.00	39.00	39.00	42.00	42.00
Soybean meal	18.00	15.00	15.00	18.00	15.00	15.00	15.00	12.00	12.00
Distiller’s dried grains with soluble, DDGS	6.00	6.00	6.00	6.00	6.00	6.00	6.00	6.00	6.00
Premix ^1^	5.00	5.00	5.00	5.00	5.00	5.00	5.00	5.00	5.00
CaCO_3_	2.00	2.00	2.00	2.00	2.00	2.00	2.00	2.00	2.00
NaHCO_3_	0.00	0.00	0.00	0.00	0.00	0.00	0.00	0.00	0.00
NaCl	1.00	1.00	1.00	1.00	1.00	1.00	1.00	1.00	1.00
NH4Cl	0.00	0.00	0.00	0.00	0.00	0.00	0.00	0.00	0.00
Total	100.00	100.00	100.00	100.00	100.00	100.00	100.00	100.00	100.00
Nutrient levels									
DE (MJ/kg) ^2^	9.12	9.25	9.25	9.12	9.25	9.25	9.09	9.19	9.19
CP	13.96	13.83	13.83	13.96	13.83	13.83	12.86	12.44	12.44
EE	3.04	3.27	3.27	3.04	3.27	3.27	3.10	3.29	3.29
NDF	30.61	28.65	28.65	30.61	28.65	28.65	30.45	28.89	28.89
ADF	14.68	15.42	15.42	14.68	15.42	15.42	14.56	14.92	14.92
Ca	0.23	0.30	0.30	0.23	0.30	0.30	0.23	0.27	0.27
P	0.23	0.23	0.23	0.23	0.23	0.23	0.22	0.22	0.22

^1^ Each kilogram of premix provided the following: Vitamin A (VA) 25,000 IU, Vitamin D (VD) 315,000 IU, Vitamin E (VE) 200.5 IU, Vitamin B2 (VB2) 116 mg, Vitamin B1 (VB1) 100.05 mg, Niacin 80 mg, Pantothenic Acid 50 mg, Biotin 0.84 mg, Folic Acid 6.4 mg, Copper (Cu) 56 mg, Iron (Fe) 550 mg, Zinc (Zn) 328 mg, Manganese (Mn) 188 mg, Iodine (I) 2.35 mg; ^2^ Digestible energy was calculated based on the ingredients of the diet and their digestible energy content and not based on the actual dry matter intake. CON: basal diet; AM_S: basal diet supplemented with Astragalus straw; FAM_S: basal diet supplemented with fermented Astragalus straw.

**Table 3:** Effects of fermented and unfermented astragalus straw on the growth performance of lambs (*n* = 9).

Items ^1^	Groups			SEM ^2^	*p*-Value
	CON	AM_S	FAM_S		
Initial weight (IBW, kg)	19.500	18.875	18.938	1.627	0.703
Final weight (FBW, kg)	49.780 ^b 3^	52.600 ^a^	53.433 ^a^	2.329	0.013
Dry matter intake (DMI, kg/d)	1.899 ^b^	1.914 ^b^	2.051 ^a^	0.085	0.003
Total weight gain (TWG, kg)	30.280 ^b^	33.725 ^a^	34.495 ^a^	2.917	0.020
Average daily gain (ADG, kg)	0.336 ^b^	0.375 ^a^	0.383 ^a^	0.032	0.020
Feed conversion ratio (F/G)	5.849 ^a^	5.144 ^b^	5.356 ^b^	0.425	0.014

^1^ IBW = Initial weight; FBW = Final weight; DMI = Dry matter intake; TWG = Total weight gain; ADG = Average daily gain; F/G = Feed conversion ratio; ^2^ SEM = standard error of the mean. ^3^ a–b different letters on the shoulder of values in a row mean a significant difference (*p* < 0.05). CON, basal diet; AM_S, basal diet supplemented with Astragalus straw; FAM_S, basal diet supplemented with fermented Astragalus straw.

**Table 4:** Effects of fermented and unfermented astragalus straw on serum antioxidant parameters in lambs (*n* = 9).

Items ^1^	Groups			SEM ^2^	*p*-Value
	CON	AM_S	FAM_S		
Superoxide dismutase (SOD, U/mL)	40.450	37.195	37.027	2.550	0.843
Catalase (CAT, U/mL)	0.894	0.765	1.034	0.097	0.524
Glutathione peroxidase (GSH-Px, U/mL)	250.77 ^b 3^	324.00 ^a^	290.26 ^a^	37.328	0.003
Malondialdehyde (MDA, nmol/g)	5.242	3.237	4.217	2.614	0.328

^1^ SOD = Superoxide dismutase; CAT = Catalase; GSH-Px = Glutathione peroxidase; Malondialdehyde = MDA; ^2^ SEM = standard error of the mean. ^3^ a–b different letters on the shoulder of values in a row mean a significant difference (*p* < 0.05). CON, basal diet; AM_S, basal diet supplemented with Astragalus straw; FAM_S, basal diet supplemented with fermented Astragalus straw.

**Table 5:** Effects of fermented and unfermented astragalus straw on serum lipid metabolism parameters in weaned lambs (*n* = 9).

Items ^1^	Groups			SEM ^2^	*p-*Value
	CON	AM_S	FAM_S		
Fatty acid synthase (FAS, U/mL)	0.560 ^a 3^	0.449 ^a^	0.380 ^b^	0.049	0.048
Acetyl-CoA carboxylase (ACC, U/mL)	15.574	14.719	14.309	5.306	0.889
Stearoyl-CoA desaturase (SCD, ng/mL)	6.623	5.249	5.544	1.369	0.136
Adipose triglyceride lipase (ATGL, ng/mL)	1298.81 ^b^	1744.00 ^a^	1703.67 ^a^	75.163	0.001
Lipoprotein lipase (LPL, mmol/L)	208.39	184.72	198.93	41.852	0.533
Total cholesterol (T-CHO, mmol/L)	0.649	0.456	0.674	0.267	0.227
High-density lipoprotein cholesterol (HDL-C, mmol/L)	0.586 ^b^	0.640 ^b^	0.697 ^a^	0.025	0.047
Non-esterified fatty acids (NEFA, mmol/L)	0.246	0.217	0.297	0.131	0.476
Low-density lipoprotein cholesterol (LDL, mmoL/L)	0.778 ^a^	0.681 ^b^	0.621 ^c^	0.054	0.001

^1^ FAS = Fatty acid synthase; ACC = Acetyl-CoA carboxylase; SCD = Stearoyl-CoA desaturase; SCD = Stearoyl-CoA desaturase; ATGL = Adipose triglyceride lipase; LPL = Lipoprotein lipase; T-CHO = Total cholesterol; HDL-C = High-density lipoprotein cholesterol; NEFA = Non-esterified fatty acids; LDL = Low-density lipoprotein cholesterol; ^2^ SEM = standard error of the mean. ^3^ a–b different letters on the shoulder of values in a row mean a significant difference (*p* < 0.05). CON, basal diet; AM_S, basal diet supplemented with Astragalus straw; FAM_S, basal diet supplemented with fermented Astragalus straw.

**Table 6:** Effects of fermented and unfermented astragalus straw on hormone levels in lambs (*n* = 9).

Items ^1^	Groups			SEM ^2^	*p*-Value
	CON	AM_S	FAM_S		
Glucagon (GC, ng/mL)	8.033 ^b 3^	11.329 ^b^	20.916 ^a^	3.709	0.001
Resistin (ng/mL)	70.068 ^a^	52.759 ^b^	43.854 ^b^	14.488	0.005
Insulin-like growth factor 1 (IGF-1, ng/mL)	0.129	0.135	0.148	0.027	0.374
Insulin (INS, μU/mL)	18.900 ^a^	14.976 ^b^	13.132 ^b^	2.853	0.002
Leptin (LEP, ng/mL)	7.228	4.778	6.808	2.518	0.139
Adiponectin (ADPN, ng/mg)	3.719	3.713	3.920	0.980	0.891

^1^ GC = Glucagon; IGF-1 = Insulin-like growth factor 1; INS = Insulin; LEP = Leptin; ADPN = Adiponectin; ^2^ SEM = standard error of the mean. ^3^ a–b different letters on the shoulder of values in a row mean a significant difference (*p* < 0.05). CON, basal diet; AM_S, basal diet supplemented with Astragalus straw; FAM_S, basal diet supplemented with fermented Astragalus straw.

## Data Availability

The data presented in this study are available within the article.

## Supplementary Materials

The following supporting information can be downloaded at https://www.mdpi.com/article/10.3390/ani16193001/s1, Figure S1: Variable importance in projection (VIP) plots coupled with abundance heatmaps of differential metabolites in serum and liver; Table S1: Alpha diversity indices of microbial communities.
