# Supplementary material for: Integrated Multi-Omics Analysis Identifies Potential Associations of Unfermented and Fermented Astragalus Straw with Serum Antioxidant Status and Lipid Metabolism in Lambs
Source: Animals (Basel). 2026 Sep 24;16(19):3001. doi: 10.3390/ani16193001 (PMC13663450; doi:10.3390/ani16193001)
Supplement: Supplementary file 1 [file animals-16-03001-s001.zip › animals-4509144-supplementary.pdf]

SupplementaryContent

Table S1. Alpha diversity indices of microbial communities.

Items	Groups			*p*-Value
	CON	AM_S	FAM_S	
Ace	503.048 ± 178.116	440.618 ± 189.389	485.765 ± 213.329	0.142
Chao 1	502.930 ± 178.129	440.678 ± 189.560	485.960 ± 213.403	0.144
Shannon	4.775 ± 1.164	4.480 ± 1.247	4.681 ± 1.250	0.198
Simpson	0.055 ± 0.095	0.068 ± 0.102	0.065 ± 0.127	0.247
Sobs	501.815 ± 178.102	439.482 ± 189.132	484.241 ± 213.101	0.142

Values are means + S.E. (n = 6). Different superscript letters in the same row indicate significant differences ($p < 0.05$).

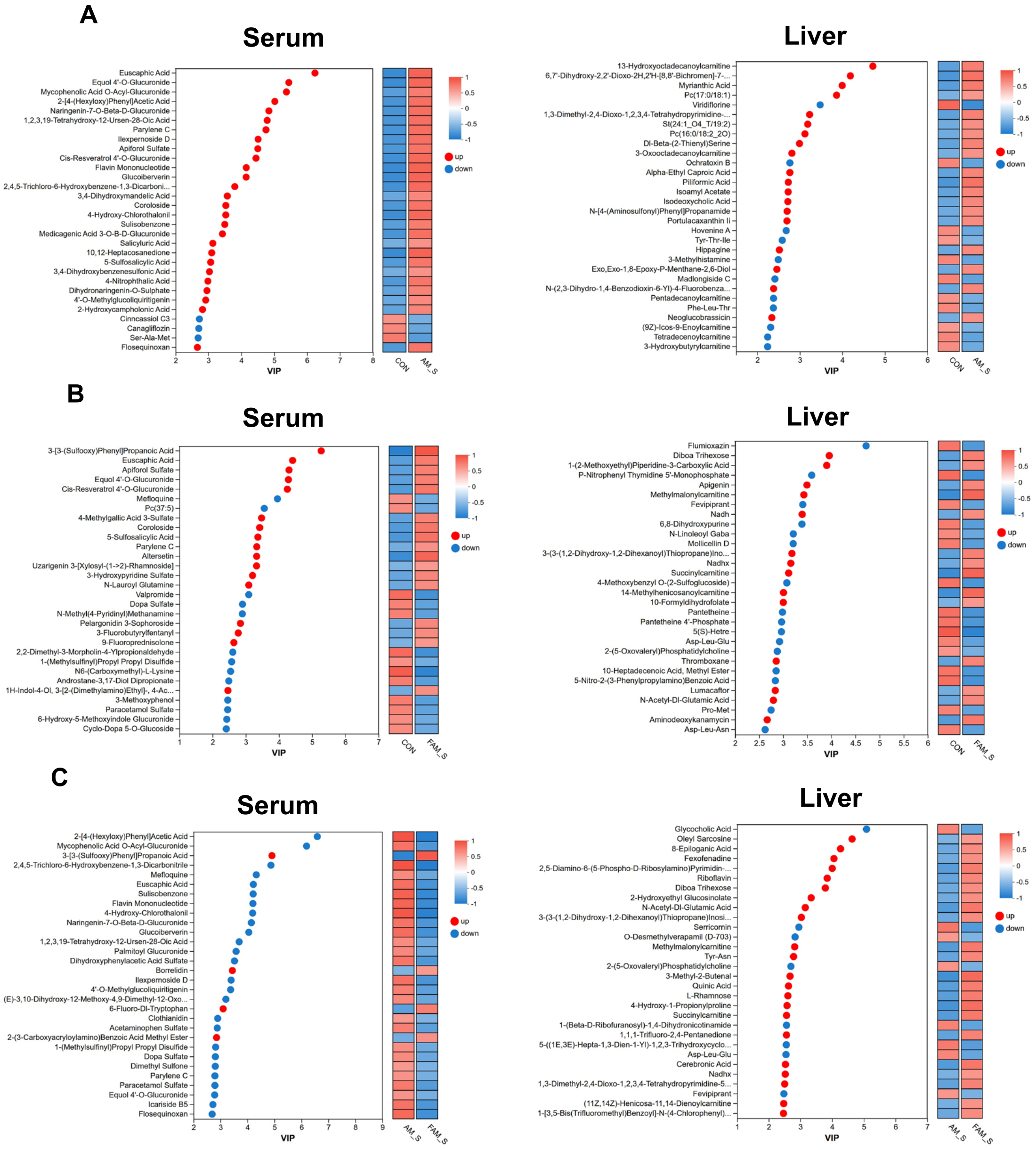

Figure S1. Variable importance in projection (VIP) plots coupled with abundance heatmaps of differential metabolites in serum and liver. (A) Differential metabolites between CON and AM_S groups; (B) Differential metabolites between CON and FAM_S groups; (C) Differential metabolites between AM_S and FAM_S groups. The horizontal axis represents VIP value. Red dots denote significantly up-regulated metabolites, and blue dots denote significantly down-regulated metabolites. The heatmaps display the relative abundance of corresponding metabolites. The basal diet (CON), the basal diet supplemented with astragalus straw (AM_S), and the basal diet supplemented with fermented astragalus straw (FAM_S).
